# 磁性共价有机骨架材料富集检测典型有机污染物的应用进展

**DOI:** 10.3724/SP.J.1123.2024.07020

**Published:** 2025-02-08

**Authors:** Liushan JIANG, Qingxiang ZHOU

**Affiliations:** 中国石油大学(北京)化学工程与环境学院,北京102249; College of Chemical Engineering and Environment, China University of Petroleum-Beijing, Beijing 102249, China

**Keywords:** 磁性固相萃取, 磁性共价有机骨架材料, 农药, 内分泌干扰物, 药物及个人护理产品, 综述, magnetic solid-phase extraction (MSPE), magnetic covalent organic framework (MCOFs), pesticide, endocrine disruptors (EDCs), pharmaceutical and personal care products (PPCPs), review

## Abstract

痕量污染物因其在环境中的微量存在和基质的复杂性,难以直接通过仪器进行检测,对人类的健康构成潜在威胁。磁性固相萃取(MSPE)技术因具有操作简单、成本低廉、富集效率高等优势,在样品前处理领域一直备受关注。磁性固相萃取的研究聚焦于磁性材料的功能化,近年来研究者深入探索了磁性材料的功能化途径,将多种新兴材料引入磁性材料的修饰,有效拓展了磁性功能化材料的性能和应用领域。其中,磁性共价有机骨架材料(MCOFs)是一种具有代表性的材料,不仅具有比表面积大、稳定性高的特点,还兼具磁性材料的快速分离特性;可通过氢键作用、疏水作用、*π-π*共轭作用实现对目标物的快速、高效富集。本文重点综述了MCOFs的种类、合成方法及其在富集检测农药、内分泌干扰物(EDCs)及药物和个人护理产品(PPCPs)中的应用,并对该领域未来研究的发展方向进行了展望。

## Abstract (en)

Trace contaminants are toxic and their widespread presence in the environment potentially threatens human health. The levels of these pollutants are often difficult to determine directly using instruments owing to the complexities of environment matrices. Hence, pretreatment steps, such as sample purification and concentration, are key along with various processes that enhance the accuracy and sensitivity of the detection method. To date, researchers have successfully developed a variety of efficient and reliable sample-pretreatment techniques that are based on different principles. Among these, magnetic solid-phase extraction (MSPE) is a rapid and efficient sample-pretreatment technology that is based on the similar solid-phase-extraction (SPE) principle, which mainly enriches target analytes by exploiting their interactions with functional groups on the surfaces of magnetic materials, thereby achieving rapid separation when an external magnetic field is applied. MSPE has been a focus of attention in the environmental-sample-pretreatment, food-analysis, and other fields owing to advantages that include ease of operation, low cost, and high enrichment efficiency. The selection of the magnetic material is key to MSPE process because traditional magnetic materials exhibit certain functionality limitations. Accordingly, designing and synthesizing green and efficient functionalized magnetic materials have become a research focus in this field, with researchers having extensively explored multiple ways of preparing functionalized modified magnetic materials through the introduction of a variety of emerging materials, including metal-organic frameworks (MOFs), covalent organic frameworks (COFs), carbon nanotubes (CNTs), graphene oxide (GO), and other specific functional groups to modify magnetic materials and effectively expanded the applications scope of MSPE. Among these, COFs are porous crystalline materials consisting of light elements (C, N, H, O and B, etc.) linked through covalent bonds. COFs are mainly classified as imine COFs, boronic-acid COFs, triazine COFs, and ketenimine COFs according to bonding type. Moreover, it is worth mentioning that COFs can be synthesized from a number of monomers, and the functional groups exposed on the COF surface can also be used for further modification purposes. COFs are versatile and modifiable; consequently, they have attracted significant research attention, with a series of COF-functionalized magnetic materials having been designed and synthesized. The magnetic COFs (MCOFs) combine the advantages of COFs and magnetic materials. MCOFs are not only endowed with the large specific surface areas, high porosities, and good stabilities that are characteristic of COFs, but also exhibit the rapid separation and reusability characteristics of magnetic material, thereby quickly and efficiently enriching targets through hydrogen bonding, hydrophobicity, *π-π* stacking, and van der Waals forces, making them ideal sample-pretreatment materials. MCOFs have also been converted into more-versatile functional materials using post-modification strategies. Combining MCOFs with advanced analytical techniques, such as high performance liquid chromatography (HPLC) and gas chromatography-mass spectrometry (GC-MS) has effectively improved the limits of detection (LODs) for various analytes as well as method accuracy; these techniques have been widely used to enrich and detect trace pollutants. However, some material design and synthesis challenges remain and need to be overcome, despite the promising potential of MCOFs. Future research needs to focus on exploring novel synthetic strategies that reduce experimental costs and improve the functionalities of MCOFs while expanding their applicabilities to broader sample matrices. In this review, we first introduce and discuss the construction and functionalized designs of various MCOF composites, and then summarize their use in applications that include the enrichment and detection of pesticides, endocrine disruptors (EDCs), pharmaceuticals, and personal care products (PPCPs), and finally provide an outlook on future developmental prospects.

随着经济的持续增长,大量污染物释放到环境中,并通过呼吸、皮肤接触以及食物链传递等多种方式进入人体,对人类健康造成潜在威胁^[1]^。目前常用的检测技术有液相色谱法^[2]^、液相色谱-质谱法^[3,4]^、超高效液相色谱-四极杆飞行时间质谱法^[5]^、气相色谱-质谱法^[6⇓-8]^等。但复杂的样品基质对于痕量污染物的检测造成很大困扰,因此样品前处理技术成为提升方法准确度的关键环节。样品前处理技术旨在降低干扰物和基质效应产生的影响,使目标物得到净化富集,达到分析仪器的检测要求,从而获得更为准确的分析效果^[9,10]^。常见的样品前处理方法主要包括固相萃取^[11]^、液液萃取^[12]^、分散液液微萃取^[13]^、分散固相萃取^[14]^、固相微萃取^[15,16]^和磁性固相萃取(MSPE)^[17⇓-19]^等。其中,MSPE是由Šafaříková等^[20]^于1999年基于SPE技术提出的一种样品预处理技术。该技术只需通过外部磁场即可实现材料的快速分离和收集,无需填充,有效缩短了分析时间^[21,22]^。对MSPE而言,磁性纳米粒子的选择至关重要。通常,磁性中心一般以铁、钴、镍及其氧化物(如磁铁矿、纳米零价铁等)、铁氧体(MFe_2_O_4_(M=Cu、Ni、Co等))和金属合金(FePt、CoPt)为主^[23⇓-25]^。但单一磁性纳米粒子容易出现团聚现象,且表面官能团较为单一,难以获得满意的萃取效果。因此,对磁性材料进行功能化修饰,提升其富集能力或选择性成为研究的热点,多种新型纳米材料如石墨烯(GO)^[11]^、碳纳米管(CNTs)^[26⇓-28]^、离子液体^[29,30]^、金属有机骨架(MOFs)^[31,32]^及共价有机骨架材料(COFs)^[33⇓-35]^等成为功能化修饰材料的主要对象。其中,COFs是一类具有有序多孔结构的晶体材料,由轻元素(H、C、B、N、O等)通过共价键聚合而成^[36,37]^,通常具有较大的比表面积、规律的孔隙、可调节的孔径大小、出色的热稳定性及化学稳定性^[38]^。同时,COFs表面的官能团为进一步的功能化修饰提供了活性位点,使构建的磁性共价有机骨架(MCOFs)复合材料在多个领域展现出独特的性能。迄今为止,研究者已基于不同配体设计合成了多种功能化MCOFs,如磁性亚胺类COFs、磁性硼酸类COFs、磁性三嗪类COFs及磁性酮烯胺类COFs等^[34,39⇓⇓⇓⇓⇓-45]^。这些磁性材料在富集痕量污染物方面表现出良好的性能,同时基于它们建立的分析方法展现出较高的灵敏度,显著提高了样品分析的准确度,并提升了其应用潜力。

基于磁性COFs在污染物富集与检测方面表现出的巨大潜力,研究者们展开了大量研究,并取得重要成果。本文归纳总结了MCOFs材料的种类、合成方法及其在富集检测农药、内分泌干扰物(EDCs)、药物及个人护理产品(PPCPs)方面的应用,并对MCOFs的应用前景及其挑战进行了展望。

## 1 MCOFs分类

磁性纳米材料表面具有的官能团相对有限,其主要通过金属的活性位点及孔隙与目标物进行作用,萃取效率较差。COFs是一类高度有序的多孔结晶聚合物,由C、H、O、N、B等轻质元素形成特殊结构,且有机结构单元可以通过共价键形成2D和3D COFs框架结构材料^[46]^。在2D COFs中,*π-π*共轭作用使其具有较大的比表面积,同时结构中形成许多1D的通道^[38,47]^。相较于2D COFs, 3D COFs具有更大的比表面积,但由于四面体结构的多样性有限,因此鲜有报道。COF的构建单元中含有*π*共轭体系、氨基和羟基官能团,其与目标物之间可以通过氢键、*π-π*共轭作用、疏水作用、静电吸引等进行相互作用^[48]^。将COF与磁性纳米材料结合构建新型功能化材料能够充分发挥两者的优势,从而扩宽材料的应用范围。根据COF的结构特征,可将MCOFs分为磁性硼酸类COFs、磁性亚胺类COFs、磁性三嗪类COFs及磁性酮烯胺类COFs等。

### 1.1 磁性硼酸类COFs

COF-1和COF-5是两种较为典型的硼酸类COFs,由Yaghi等^[49]^分别通过苯硼酸分子脱水缩合、苯硼酸与2,3,6,7,10,11-六羟基甲苯(HHTP)脱水缩合而得。两类COFs均具有较大的比表面积,能够为目标物提供较多的吸附位点^[50]^。2017年,Chen等^[34]^利用仿生聚多巴胺功能化法将获得的COF-1固定在磁性纳米颗粒表面,成功合成了MCOFs复合材料(M-COF-1),并将其应用于萃取大鼠血浆中的紫杉醇。Wang等^[43]^采用自组装技术将COF-5与磁性石墨烯复合,获得了COF功能化的磁性石墨烯生物复合材料(MagG@COF-5)。磁性石墨烯的*π-π*电子共轭结构特性和强磁性不仅为目标物提供了丰富的亲和位点,还增强了COF层的吸附能力。同时,COF-5的介孔结构(孔径约为1.8 nm)排除了复杂生物样品中大尺寸的干扰物。基于此,该材料在处理超低量的人血清样品(1 μL)时对*N*-链糖肽展现出高效的富集效率。但硼元素的缺电子性质使硼酸类COFs对亲核试剂的进攻十分敏感,导致此类COFs的稳定性相对较弱。因此,有关磁性硼酸类COFs用于磁性固相萃取环境中污染物的报道相对较少。

### 1.2 磁性亚胺类COFs

亚胺类COFs是一类由有机单体通过席夫碱反应(Schiff-base reaction)缩合而成的多孔有机材料^[38]^。相较于硼酸类COFs,亚胺类COFs具有更高的稳定性,能够在水、常用有机溶剂以及酸、碱溶液中稳定存在^[51]^。亚胺类COFs的合成条件较为简单且配体种类丰富,提供氨基的配体主要有1,3,5-三(4-氨基)苯基苯(TAPB)^[52]^、对苯二胺^[53,39]^、联苯胺(BD)^[54⇓-56]^及1,3,5-三(4-氨基苯基)三嗪(TAPT)^[57]^等;而提供醛基的配体主要包括对苯二甲醛(TPA)^[58,59]^、1,3,5-均苯三甲醛(TFB)^[60,39]^、2,5-二羟基-1,4-苯二甲醛(Dt)^[61]^及2,5-二乙烯基苯甲醛(Dva)^[62,63]^等。鉴于配体结构和性质上的差异,磁性材料可以与多种配体进行结合进而获得多种具有不同功能的磁性复合材料。Deng等^[58]^将TAPB、TPA及水热合成的Fe_3_O_4_分散在二甲基亚砜(DMSO)中,加入乙酸静置后得到具有核壳结构的Fe_3_O_4_@COFs,复合材料的壳层厚度约为25 nm,合成的MCOFs表面富含氨基,显著提升了材料的极性及亲水性,同时基于氢键以及*π-π*共轭作用,MCOFs对4-*n*-壬基酚、4-*n*-辛基酚、双酚A和双酚AF表现出良好的富集能力。Xu等^[64]^以乙腈为溶剂,利用乙酸催化2,3,5,6-四氟对苯甲醛(TFTA)和TAPT在羧基功能化Fe_3_O_4_表面进行原位聚合,成功获得具有海胆结构的含氟磁性共价有机骨架Fe_3_O_4_@TAPT-TFTA-COF(图1a~d)。基于良好的氟亲和力,Fe_3_O_4_@TAPT-TFTA-COF对含氟的苯甲酰脲类杀虫剂表现出较强的选择性和富集能力。如图1e所示,Li等^[65]^通过TPA与TAPB在Fe_3_O_4_表面的缩合反应成功制备了高效去除三氯生(TCS)和三氯卡班(TCC)的磁性吸附剂Fe_3_O_4_@COFs,该吸附剂在去除生物样品中的三氯生和三氯卡班方面表现出较高的稳定性和优异性能。该吸附剂对胎牛血清样品中三氯生和三氯卡班的去除率和回收率分别为82.3%~95.4%和92.9%~109.5%,同时Fe_3_O_4_@COFs在10次应用循环后仍能保持良好的稳定性,具有较强的再生能力。

**图1:**
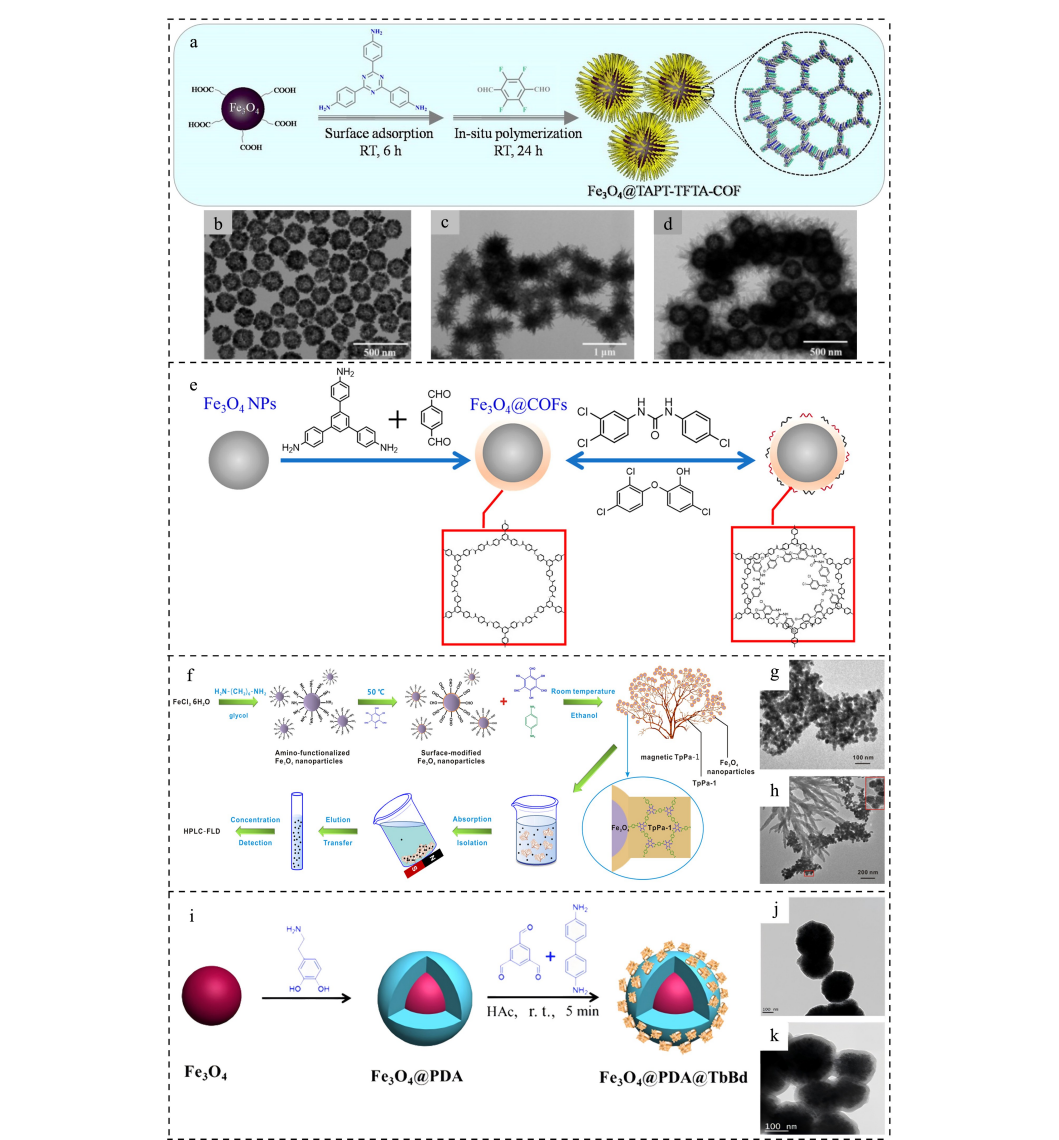
(a)Fe_3_O_4_@TAPT-TFTA-COF的合成机理图^[64]^; (b)Fe_3_O_4_、(c)TAPT-TFTA-COF和(d)Fe_3_O_4_@TATP-TFTA-COF的 透射电镜图^[64]^; (e)Fe_3_O_4_@COFs的合成及高效吸附去除TCS和TCC^[65]^; (f)花状磁性TpPa-1的合成及应用^[53]^; (g)NH_2_-Fe_3_O_4_和(h)磁性TpPa-1的透射电镜图^[53]^; (i)Fe_3_O_4_@PDA@TbBD的合成路线图^[69]^; (j)Fe_3_O_4_@PDA和(k)Fe_3_O_4_@PDA@TbBD的透射电镜图^[69]^

### 1.3 磁性三嗪类COFs

三嗪类COFs(CTFs)是一类由1,3,5-三嗪环通过三嗪键聚合而成的有机聚合物,由于苯环和三嗪环之间的共轭作用降低了有机骨架中*π*共轭分子的总能量,因此该类COFs表现出较高的化学稳定性^[66]^。Zhang等^[41]^以FeCl_3_·6H_2_O和1,4-二氰基苯(DCB)为原料,利用微波-增强高温离子热法制备了一系列不同*γ*-Fe_2_O_3_含量的磁性复合物CTF/Fe_2_O_3_。合成材料的比表面积可达930~1149 m^2^/g, CTF/Fe_2_O_3_(Fe_2_O_3_含量为11.14%)能够快速吸附水溶液中的甲基橙,吸附容量为291 mg/g。Ma等^[67]^将同样方法制得的磁性CTF成功用于真实水样中多种苯酚类化合物的富集分析,获得的加标回收率为85.5%~97.7%。2022年,Wang等^[42]^将CTF和Fe_3_O_4_混合,采用球磨法成功合成出具有不同CTF/Fe_3_O_4_质量比的磁性复合材料,用于吸附去除全氟壬烯氧苯磺酸钠和六氟环氧丙烷三聚酸,吸附容量分别为1.18和1.02 mmol/g。

### 1.4 磁性酮烯胺类COFs

酮烯胺类COFs是一类主要由2,4,6-三甲酰基间苯三酚(Tp)与含有芳香胺的配体先后通过席夫碱反应及不可逆的质子互变异构反应形成的有机多孔材料。由于具有高结晶度和出色的水稳定性,研究者经常将酮烯胺类COFs与磁性材料相结合构建磁性吸附剂。2017年,He等^[53]^报道了一种萃取多环芳烃(PAHs)的花状MCOFs(图1f),该材料由氨基功能化的Fe_3_O_4_(NH_2_-Fe_3_O_4_)、Tp与对苯二胺(Pa-1)缩合形成的多孔COF纳米纤维组成(图1g和图1h)。由于TpPa-1骨架中具有高比例的N和O原子以及*π-π*离域体系,仅5 mg MCOFs即可实现对6种PAHs的快速富集。Shi等^[68]^利用Tp和2,6-二氨基蒽醌(DA)在Fe_3_O_4_表面发生的缩合反应获得具有高比表面积及多孔性的磁性酮烯胺类COF(Fe_3_O_4_@COF),基于*π-π*共轭和疏水作用,其对15种PAHs表现出优异的萃取能力。Yan等^[69]^将Tp、BD及聚苯胺预修饰的Fe_3_O_4_分散在DMSO中,加入乙酸,室温静置后获得了具有核壳结构的亲水性磁性纳米球Fe_3_O_4_@PDA@TbBd(图1i~k)。该材料不仅具有独特的*π-π*电子体系、良好的亲水性和磁性,且具有规则的多孔结构和较大的比表面积。基于这些优势,Fe_3_O_4_@PDA@TbBd被用于富集检测血浆中的邻苯二甲酸酯(PAEs),且成功检出9种PAEs。2024年,Yao等^[45]^将Tp与3,3'-二羟基联苯胺(DHBD)混合研磨后得到的COF(Tp-DHBD)分散在含有铁离子的溶液中,调至碱性后,80 ℃下反应合成了对12种有机磷酸酯具有较好富集性能的Fe_3_O_4_/Tp-DHBD。

### 1.5 功能化MCOFs

由于单体COFs的官能团相对单一,MCOFs表面可提供的功能活性位点较为有限,导致材料在萃取复杂基质中的多种污染物时面临较大的挑战。因此,需要对MCOFs进行功能化修饰,以克服这一局限性。目前,功能化修饰磁性COFs的方法主要分为前修饰策略和后修饰策略,前者是利用功能化分子或通过修饰衍生的单体直接构建功能化COFs,而后者是将功能化分子接枝在已经合成的COFs表面^[36]^。相较于前修饰策略,后修饰策略不仅能保证材料结构的稳定性,还可通过酯化反应及“点击”反应等对材料表面的官能团进行转化,引入设计的亲/疏水性、酸/碱性或含S、N等的官能团改变材料的孔隙结构、表面电荷分布及亲疏水特性,从而提升材料与目标物的相互作用力,因此后修饰策略获得了广泛应用。Gao等^[70]^以具有高磁响应的磁性胶体纳米晶团簇(MCNCs)为磁性中心,以1,3,5-三(4-甲酰基联苯)苯(TfPb)和3,3'-二羟基联苯胺(DHBD)缩合形成二维COF壳层,通过连续后合成策略将Zr^4+^离子固定在COF表面,成功制备了具有可控核壳结构的MCNC@COF@Zr^4+^(图2a)。高负载量的Zr^4+^使MCOFs对磷酸肽表现出良好的吸附能力以及高选择性。Luo等^[71]^首先采用TAPB和2,5-二乙烯基对苯二甲酸在MCNC表面构建COF壳层,随后通过巯基-烯的“点击反应”将谷胱甘肽(GSH)修饰到COF壳层上,获得具有快速磁响应、大比表面积和良好亲水性的磁性微球MCNC@COF@GSH(图2b)。所制备的MCN@COF@GSH对唾液中的*N*-连接糖肽表现出卓越的富集性能和尺寸排阻效果,成功检出唾液中143种内源性*N*-连接糖肽。同年,Gao等^[72]^通过叠氮化合物与炔烃之间的“点击”反应成功合成了具有苯硼酸修饰的磁性共价有机骨架MCNCs@COF@PBA (图2c),用于特异性捕获糖蛋白和蛋白质中的*N*-连接肽,同时进行蛋白质排斥。以MCNCs@COF@PBA为吸附剂,结合MS建立的方法成功用于分析海拉细胞分泌的外泌体中的糖肽,并表现出较高的灵敏度和选择性。Wang等^[73]^利用原位生长策略将溴化卟啉COF包裹在Fe_3_O_4_-NH_2_纳米球的表面,通过Suzuki-Miyaura反应将硼酸引入到COF中得到具有核壳结构的硼酸功能化磁性卟啉基共价有机骨架材料(Fe_3_O_4_@BA-COF)。卟啉基COF富氮骨架提供的亲水作用和硼酸基团赋予的硼亲和力使材料对含顺式二醇的核苷如腺苷、鸟苷、儿茶酚及槲皮素均具有良好的富集效果。

**图 2:**
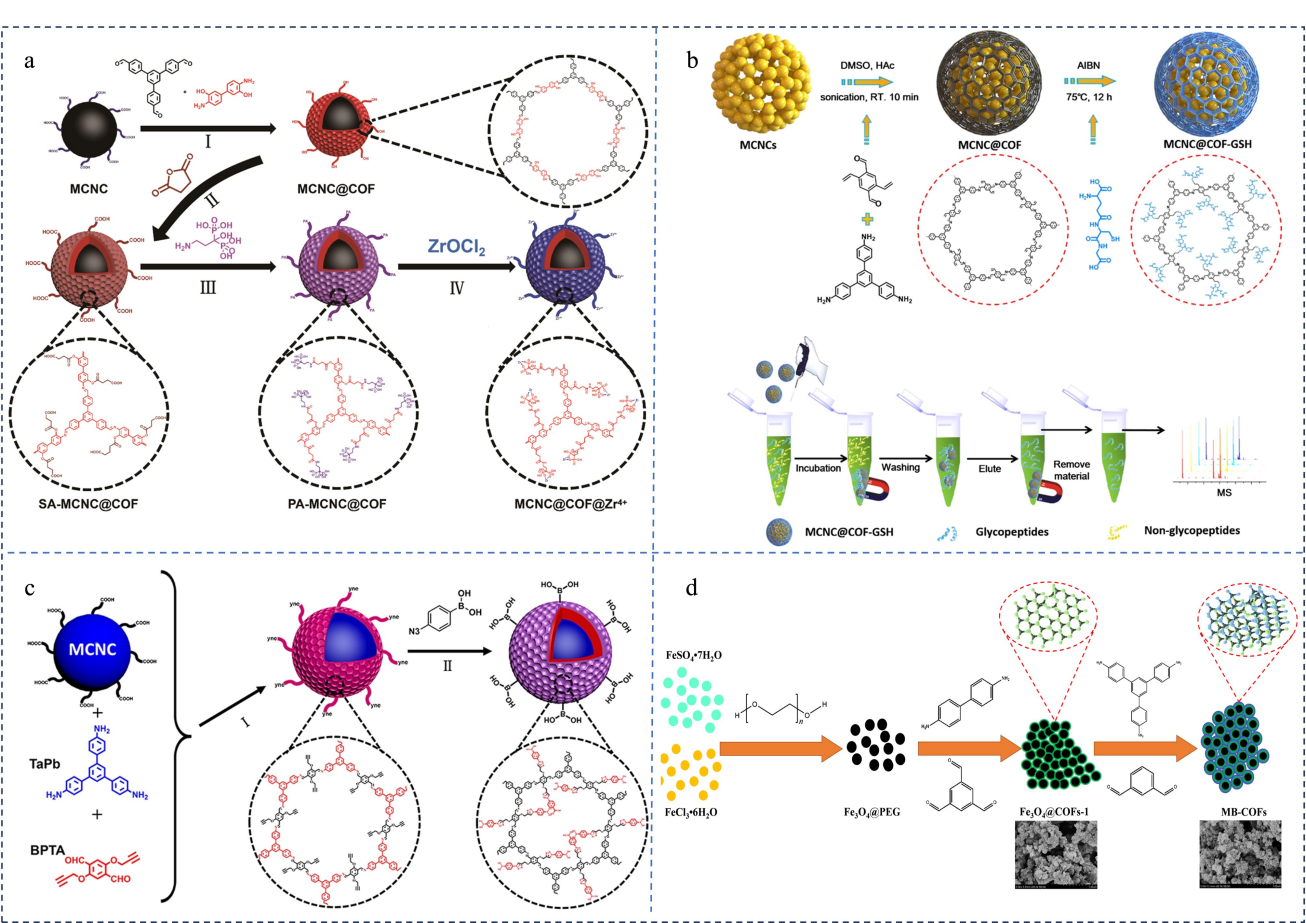
(a)顺序合成后修饰法制备Zr^4+^固定化磁性COF的机理图^[70]^; (b)MCNC@COF-GSH微球的合成示意图^[71]^; (c)MCNCs@COF@PBA的合成及磁性富集糖肽的示意图^[72]^; (d)球形团簇结构MB-COF的合成示意图^[75]^

除有机分子和金属离子外,不少研究者还利用MOFs、COFs、碳材料及过渡金属碳氮化合物(MXene)等对MCOFs进行修饰,构建功能性更强的磁性纳米材料。环三甲苯基COF(CTC-COF)是一种由碗状CTC单体通过硼酸酯键连接而成的共价有机聚合物,虽然具有较大的比表面积,但因容易受到亲核试剂的影响,导致材料的应用受到限制。Liang等^[74]^将该COF与装有磁性纳米粒子的疏水性碳纳米管结合,有效增强了COF的水稳定性,并将合成的复合材料(CTC-COF@MCNT)成功用于萃取分离食品中的杂环芳香胺。Zhou等^[75]^通过单体介导原位生长策略和逐层组装的方法成功制备了COF-on-COF修饰的磁性材料(MB-COF),并用于快速吸附和定量十字花科蔬菜中的萝卜硫素(图2d)。该材料表面呈球形团簇结构,表面粗糙、介孔丰富且具有良好的稳定性。基于双层COF的增强作用,MB-COF相较于单一COF对萝卜硫素的吸附效率提高约18%~72%。除了提升COF对目标物的亲和力外,COF与磁性粒子之间的稳定性也一直备受关注。MXene是一种具有特殊结构的过渡金属碳/氮化合物,表面大量的负电荷基团(如-OH, -F及=O等)赋予了材料良好的亲水特性及可修饰性。基于材料的独特性质,我们课题组^[76]^将其作为磁性纳米粒子与COF之间的“纽带”,利用负电荷基团与金属离子之间的静电吸引作用获得具有高磁性响应的磁性Ti_3_C_2_,然后通过三氨丙基三乙氧基硅烷(APTES)对磁性MXene进行修饰,将修饰得到的氨基与COF配体的醛基缩合,成功制备了具有高稳定性和高富集能力的磁性复合材料CoFe_2_O_4_@Ti_3_C_2_@COF-LZU1。

## 2 MCOFs的合成方法

### 2.1 溶剂热法

溶剂热法指的是在特定温度和压力下,磁性纳米颗粒、COF单体及有机溶剂在密闭容器中进行反应获得MCOFs的方法。在该方法中,溶剂对于COFs的形成起着较为重要的作用,能够对产物的粒度、形状等特征进行控制。目前,可用于合成的溶剂主要有1,4-二氧六环^[77]^、四氢呋喃(THF)^[78]^、二甲基亚砜^[79]^、正丁醇-1,2-二氯苯(1∶1, v/v)^[52]^、1,4-二氧六环-均三甲苯(2∶3, v/v)^[80]^等。2020年,Li等^[77]^曾采用该方法成功制备了具有核壳结构的Fe_3_O_4_@COF(TpDA),并用于萃取水果中的植物生长调节剂。具体步骤如下:首先,将Tp、DA及预先合成的Fe_3_O_4_分散在1,4-二氧六环中。然后,将混合物转移至安瓿瓶,在液氮浴中迅速冷却,抽真空至19 mbar,并用火焰密封。待温度降至室温后,放置在120 ℃的烘箱中反应3天,经*N*,*N*-二甲基甲酰胺(DMF)、THF洗涤干燥后得到Fe_3_O_4_@COF(TpDA)。Zhang等^[80]^以Fe_3_O_4_、Tp及硫代卡巴肼为原料,采用溶剂热法合成了一种由肼连接且具有核壳结构的磁性复合物Fe_3_O_4_@COF-TCH。该材料表面富含亚胺和硫酮基团,对铅和汞表现出良好的吸附能力。Zheng等^[79]^将Fe_3_O_4_超声分散在含有三聚氰胺和4,4-联苯二甲醛的DMSO溶液中,然后在180 ℃下反应24 h得到对Ag纳米粒子具有较高富集能力的Fe_3_O_4_@BM。

### 2.2 浸渍沉淀法

浸渍沉淀法是指将预先合成的COFs浸入含有Fe^2+^和Fe^3+^的溶液中,浸没一段时间后,再将其转移至氨水中以促进磁性粒子的形成。2020年,Mi等^[33]^将羟基化COF浸没在含有FeSO_4_·7H_2_O与Fe_2_(SO_4_)_3_的混合溶液中,30 min后进行离心分离并将得到的产物立即转移至体积分数为0.625%的氨水中,调整pH至10~12,获得了具有一定磁响应能力的复合材料OH-MCOF。Zhuang等^[81]^将水热合成的花状COF浸没在Fe_2_(SO_4_)_3_水溶液中,4 h后通过膜分离法得到湿性COFs,然后将其转移至2.5%的氨水中,在120 ℃下反应形成Fe_2_O_3_@COFs。虽然浸渍沉淀法较为简单,且形成的磁性粒子大多存在于COF的孔道中,不会显著影响其纯度。但是该方法很难保证磁性粒子的均匀分布,导致合成的产物磁性并不强。

### 2.3 微波辅助合成法

微波辅助合成法是一种在分子水平上通过微波段电磁波实现内加热的方法,具有效率高、耗时短等优势。2011年,Zhang等^[41]^以DCB和FeCl_3_·6H_2_O为前驱体,ZnCl_2_为路易斯酸催化剂和微波吸收剂,在500~550 ℃下微波辐射1 h,成功合成了一系列具有高比表面积的CTF/Fe_2_O_3_复合物。2016年,Ge等^[82]^以三聚氰胺、对苯二甲醛和Fe_3_O_4_为原料,采用微波辅助合成法制备了选择性吸附Hg^2+^的磁性三聚氰胺基COF。具体过程是先将三聚氰胺和对苯二甲醛溶解在DMSO中,然后向内加入氨基功能化Fe_3_O_4_,混合均匀后再将溶液放置在微波炉中,280 W微波辐射下加热4 h。产物经布氏漏斗过滤后依次用丙酮、二氯甲烷、四氢呋喃洗涤,最后在120 ℃下真空干燥5 h得到Fe_3_O_4_/M-COFs。虽然该方法操作较为简单,但过快的反应速率容易导致材料的结晶性较差,难以按照预期生长,具有一定局限性。

### 2.4 机械研磨法

机械研磨法,即在无溶剂或少量溶剂下,通过研磨固体进行反应的一种合成方法。该方法避免了高温反应及有机溶剂的过度消耗,且短时间内能够实现规模化制备MCOFs,因而得到广泛使用。Lin等^[83]^采用无溶剂研磨法制备了一种能够萃取喹诺酮的磁性复合物NiFe_2_O_4_@COF。具体制备过程是先将Tp和2,2'-联苯胺二磺酸 (BD-(SO_3_)_2_)混合并研磨成灰白色粉末,然后加入NiFe_2_O_4_,研磨均匀后再加入乙酸钠,10 min后将研磨得到的浅棕色粉末转移至刚玉坩埚中,150 ℃下反应1 h,依次经过超纯水和无水乙醇洗涤及真空干燥后得到最终产物。该方法的产率约为81%,合成的材料具有良好的磁响应能力,能够在10 s内实现快速分离。Niu等^[84]^将氨基预修饰的Fe_3_O_4_、Tp和三聚氰胺加入到球磨机中,研磨5.75 h后经过磁分离得到具有折叠片状结构的Fe_3_O_4_@TpMA,磁化强度为9.28 emu/g。Liu等^[85]^首先将对甲苯磺酸(PTSA)、1,4-苯二胺(Pa-1)和磁性碳纳米管(MCNTs)加入到研钵中,研磨均匀后加入Tp,继续研磨10 min后再向混合物中逐滴加入去离子水,并研磨至黏土状,随后将混合物放置在170 ℃烘箱中加热5 min。待产物降至室温后将其研磨成粉末,经过洗涤干燥后得到的黑棕色固体即为磁性MCNTs@TpPa-1。合成的材料在水溶液中具有良好的分散性,其磁化强度为15 emu/g,能够在60 s内被磁铁分离。虽然研磨法相较于其他方法具有合成时间短、有机溶剂消耗量少的优势,但是经该方法获得的材料结晶度和孔隙度并不高^[86]^。

## 3 磁性共价有机骨架材料的应用

### 3.1 农药

在农业领域,农药对防治病虫害、增加作物产量起着关键性作用,但农药的不当使用易造成残留超标,引发食品安全和环境污染等问题。因此,环境中农药的残留监控非常重要。磁性固相萃取作为一种高效的样品前处理技术,在农药残留分析方法的开发中得到了广泛应用^[87]^。如图3a所示,Guo等^[88]^通过单体介导原位生长策略获得了具有核壳结构的乙烯基磁性共价有机骨架(Fe_3_O_4_@v-COF)。该材料表面丰富的共轭体系及氰基基团为目标物提供了较多的作用位点,以Fe_3_O_4_@v-COF为吸附剂结合HPLC成功建立了富集检测水果和蔬菜中苯并咪唑类杀菌剂的方法,建立的方法在0.2~200 μg/L范围内呈现出良好的线性,检出限为0.05~0.25 μg/L。Fan等^[89]^将对苯二甲醛和三聚氰胺缩合合成了SNW-1,进而与新型聚乙二醇(PEG-600)修饰的磁性颗粒共沉淀制备了磁性吸附剂PEG/Fe_3_O_4_@SNW-1,合成的磁性吸附剂对5种苯甲酰脲类农药具有良好的吸附能力,结合高效液相色谱建立了分析环境水样中苯甲酰脲农药的方法,方法的检出限和定量限(LOQ)分别低于1和3.4 μg/L。Chen等^[90]^以Tp和PDA为原料通过无溶剂法制备了磁性共价有机骨架材料,并基于磁性固相萃取与扫集胶束电动色谱法(MEKC)联用建立了富集检测水和大气颗粒样品中9种有机磷农药的方法。该方法的富集因子可达1740~3626,检出限为0.78~2.33 μg/L。Guo等^[91]^报道了基于苯并咪唑基磁性COF(M-TpDAB)的磁性固相萃取与液相色谱-紫外检测器(LC-UV)联用富集检测苯脲类除草剂的方法。该方法在0.15~100 ng/mL和1~100 ng/mL内分别对水样和茶叶样品中的苯脲类除草剂呈现出良好的线性,两种样品中除草剂的加标回收率分别为84.6%~105%和80.3%~102%,检出限分别为0.05~0.15和0.3~0.5 ng/mL。Tian等^[92]^制备了具有氨基功能化修饰的磁性共价有机骨架复合材料TpBd-(NH_2_)_2_@Fe_3_O_4_,丰富的孔隙结构、苯环和氨基及大比表面积使该材料对磺酰脲类除草剂表现出较高的萃取效率。基于TpBd-(NH_2_)_2_@Fe_3_O_4_建立的MSPE-HPLC-UV检测方法对磺酰脲类杀虫剂的富集因子达217~260,环境水样、土壤和烟叶样品中5种磺酰脲类除草剂的加标回收率分别为90.7%~104%、70.7%~99%、59.3%~97.8%。Yu等^[54]^采用溶剂热法合成了磁性吸附剂COF-SiO_2_@Fe_3_O_4_,与GC-MS/MS相结合建立了富集检测蔬菜(黄瓜、莴苣和白菜)中拟除虫菊酯的方法(图3b)。在最佳条件下,蔬菜中拟除虫菊酯的加标回收率为80.2%~116.7%,方法的检出限和定量限分别为0.3~1.5 μg/kg和0.9~4.5 μg/kg。

**图3:**
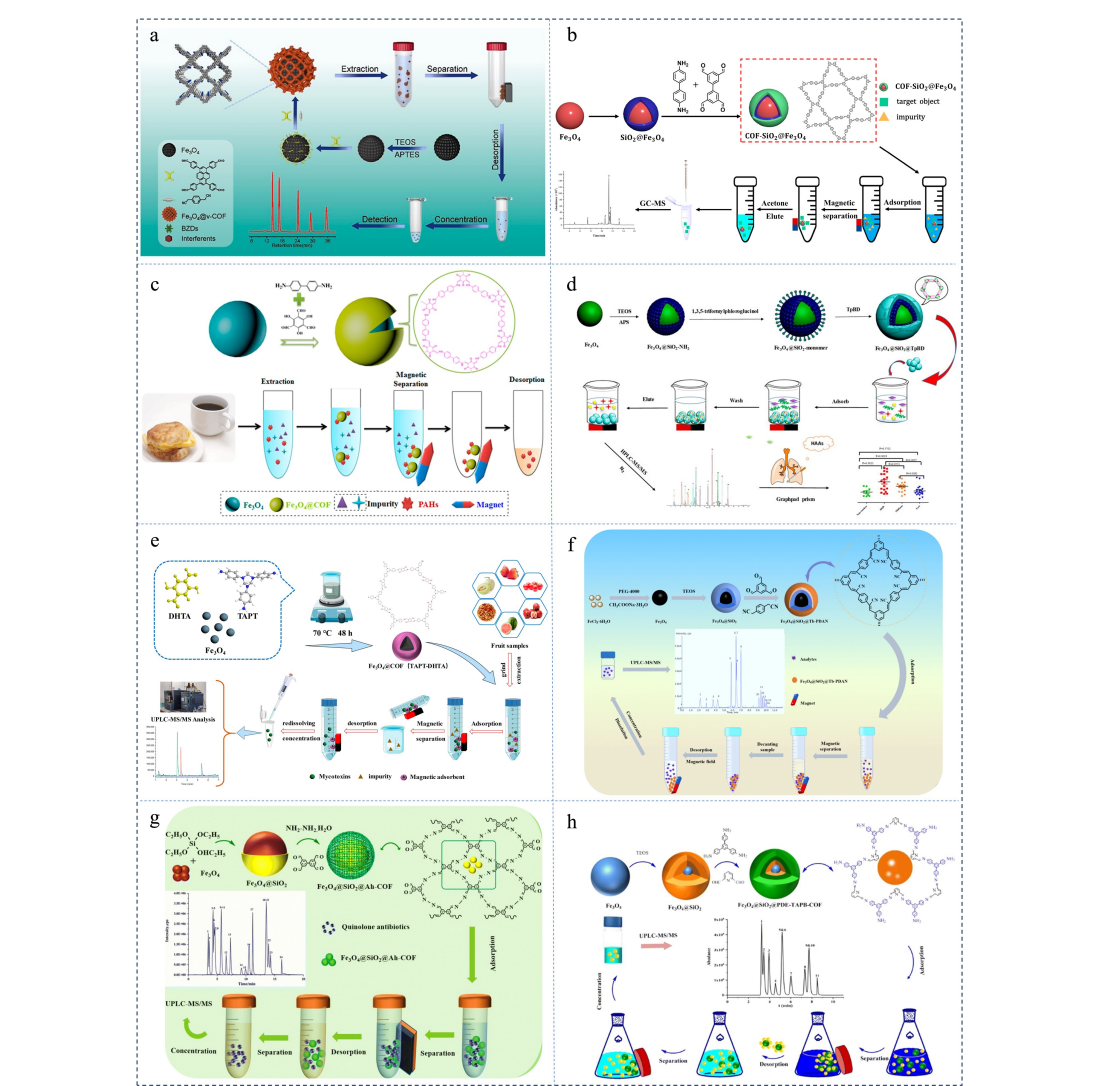
(a)Fe_3_O_4_@v-COF的合成及磁性固相萃取苯并咪唑类杀虫剂^[88]^; (b)COF-SiO_2_@Fe_3_O_4_的合成及检测拟除虫菊酯类农药^[54]^; (c)Fe_3_O_4_@COF-(TpBD)的合成及磁性固相萃取多环芳烃的示意图^[93]^; (d)Fe_3_O_4_@SiO_2_@COF-(TpBD)的合成及磁性固相萃取杂环芳香胺流程图^[94]^; (e)Fe_3_O_4_@COF(TAPT-DHTA)的构建及磁性固相萃取水果中霉菌毒素^[95]^; (f)Fe_3_O_4_@SiO_2_@Tb-PDAN的制备及磁性固相萃取MALs^[97]^; (g)Fe_3_O_4_@SiO_2_@Ah-COF的合成及富集检测喹诺酮类抗生素^[101]^; (h)Fe_3_O_4_@SiO_2_@PDE-TAPB-COF的合成及磁性固相萃取磺胺类抗生素^[103]^

### 3.2 内分泌干扰物

EDCs是一种外源性化合物,主要包括多环芳烃、邻苯二甲酸酯、多溴联苯醚及酚类等化合物,其在人体内富集后会对内分泌系统造成干扰进而威胁健康。2017年,Wang等^[60]^将COF-LUZ1固定在聚乙烯亚胺功能化修饰的磁性纳米粒子表面,获得可用于萃取多环芳烃的磁性固相吸附剂COF-LZU1@PEI@Fe_3_O_4_。基于该材料建立的MSPE-HPLC方法成功用于测定环境样品中6种多环芳烃,水样和土壤中多环芳烃的回收率分别为90.9%~107.8%和85.1%~105%。Li等^[93]^在室温条件下合成了对PAHs具有优异吸附能力的磁性COFs(Fe_3_O_4_@COF-(TpBD)),并将该材料与HPLC-DAD结合建立了富集检测15种PAHs的分析方法(图3c)。该方法的LOD为0.83~11.7 ng/L,具有较高的灵敏度。基于该方法,多种实际样品(如咖啡样品、烟熏培根、熏猪肉、野生鱼、烤鱼、咖啡和水)中存在的多环芳烃均被成功检出。Zhang等^[94]^基于Fe_3_O_4_、TP和BD构建了具有良好分散性和稳定性的双壳层磁性纳米球(图3d),并采用尿液样品中的14种杂环芳香胺对材料的富集能力进行评价。在最佳条件下,Fe_3_O_4_@SiO_2_@TpBD对14种杂环芳香胺的回收率可达95.4%~129.3%。合成的磁性纳米球材料具有良好的稳定性,经乙腈和水洗涤后,可重复利用,且性能并无显著差异。Wang等^[95]^通过模板沉淀聚合法制备了具有核壳结构的磁性共价有机骨架材料Fe_3_O_4_@COF (TAPT-DHTA)(图3e)。由于材料结构中存在丰富的羟基和芳香环,该材料对9种霉菌毒素表现出较强的富集能力。研究者以Fe_3_O_4_@COF (TAPT-DHTA)为磁性吸附剂,结合UHPLC-MS/MS建立了富集检测水果中霉菌毒素的分析方法,方法的检出限为0.01~0.5 μg/kg。Zhang等^[40]^将具有高比表面积的磁性COF(Fe_3_O_4_@TAPB-DVA)用于颗粒物中超痕量邻苯二甲酸酯的磁性固相萃取分离,并采用HPLC-MS/MS进行分析,检出限低至0.01~0.5 ng/L,同时在细颗粒物(PM_2.5_)中成功检出痕量邻苯二甲酸二(2-甲氧基乙基)酯、邻苯二甲酸二(2-正丁氧基乙基)酯、邻苯二甲酸丁苄酯和邻苯二甲酸二环己酯。2023年,Wang等^[96]^将席夫碱反应合成的COF(TFP-NDA)加入到含有铁离子的水溶液中,通过共沉淀法得到孔径尺寸为22 nm的磁性COF。合适的孔隙结构、*π-π*共轭和疏水作用使BPA和PAEs在12 min内快速达到吸附平衡,且该材料对富集检测饮料中的PAEs和双酚A(BPA)表现出很强的适用性;基于TFP-NDA/Fe_3_O_4_与气相色谱-火焰离子检测器(GC-FID)建立的分析方法呈现出较宽的线性范围和较低的检出限。Liang等^[97]^报道了一种含有氰基和C=C共轭结构的磁性吸附剂Fe_3_O_4_@SiO_2_@Tb-PDAN,并基于该材料建立了用于富集检测水和蜂蜜中16种大环内酯类抗生素(MALs)的MSPE-LC-MS/MS方法(图3f),该方法的检出限分别为0.001~0.012 μg/L和0.001~0.367 μg/L,回收率分别为71.03%~117.2%和70.02%~118.9%。

### 3.3 药物及个人护理品

PPCPs是一类新兴环境污染物,具有较强的环境持久性、生物活性和生物积累性等特点,长期暴露对环境和人体健康可造成潜在危害^[98]^。因此,开发富集检测环境中PPCPs的方法显得尤为重要。2023年,Ning等^[99]^利用原位生长策略制备了基于卟啉的新型磁性共价有机骨架Fe_3_O_4_@NH_2_@PCOF,并用于富集水样中的8种PPCPs。分子动力学模拟、独立梯度模型分析以及相关表征证明COF层中含N、O和苯基的官能团增强了Fe_3_O_4_@NH_2_@PCOF对PPCPs的吸附性能和选择性。基于该材料建立的MSPE-HPLC方法在1~500 ng/mL内表现出良好的线性范围,且具有较低的检出限,被成功用于检测废水和游泳池水中的对羟基苯甲酸酯和紫外线过滤剂。Huang等^[100]^以Pa和Tp为原料采用无溶剂法合成了一种磁性COF,基于大比表面积及MCOF与双氯芬钠之间的*π-π*堆积和疏水作用,MCOF对双氯芬钠表现出极好的萃取能力。在最佳条件下,基于MSPE与HPLC-UV/MS建立的方法呈现出较宽的线性范围(0.05~1000 μg/kg)和较低的检出限(10 ng/kg)。Li等^[101]^以SiO_2_包覆的Fe_3_O_4_为磁性中心,3,3',5,5'-四醛基联苯和水合肼(85%)为单体构建了具有核壳结构的磁性吸附剂Fe_3_O_4_@SiO_2_@Ah-COF(图3g)。基于*p*-*π*、*π-π*相互作用和氢键的协同作用,Fe_3_O_4_@SiO_2_@Ah-COF对24种喹诺酮类药物表现出良好的吸附能力。在最佳条件下,以该材料为吸附剂结合UPLC-MS/MS建立的方法在0.2~200 μg/kg表现出良好的线性关系。在水样和鸡蛋样品中24种喹诺酮的LOD分别为0.003~0.036和0.009~0.272 μg/L,加标回收率分别为70.3%~106.1%和70.4%~119.7%。2023年,Gao等^[102]^采用多元合成策略与后修饰法制备了具有羧基功能化的磁性共价有机骨架(Fe_3_O_4_@iCOF-COOH),结合HPLC-UV建立了富集检测牛奶中6种氟喹诺酮类药物(FQ)的方法。牛奶中FQ的加标回收率为68.4%~105%。Ma等^[103]^以Fe_3_O_4_@SiO_2_@PDE-TAPB-COF为磁性吸附剂,结合超高效液相色谱建立了富集检测水和食品中11种磺胺类抗生素的方法(图3h )。在最佳条件下,该方法在0.1~100 μg/L范围内表现出良好的线性,LOD为0.007~0.024 μg/L。在实际水样和鸡蛋样品中11种磺胺类抗生素的加标回收率分别为74.3%~107.2%和75.1%~102.5%,相对标准偏差低于9.56%。

## 4 结论与展望

MCOFs材料具有结构可设计、比表面积大、可修饰等优点,以功能化MCOFs材料开发磁性固相萃取技术,能够拓展其适用范围,提高萃取效率。已设计开发的多种MCOFs对农药、内分泌干扰物、药物及个人护理产品等展现了突出的吸附能力,同时结合检测技术建立的分析方法的灵敏度及准确度获得了显著改善。但新污染物的出现对磁性COFs的结构要求发生了新变化,同时对MCOFs的开发应用提出了新的发展方向:(1)目前COFs的单体设计和合成方法可能不足以满足新型污染物的需求。为了适应新污染物的检测要求,需要加强特征性COF的单体设计合成,重构合成路线,从而降低合成成本并缩短合成时间,提高材料的经济性和应用效率;(2)现有的修饰策略可能未能实现COFs与磁性材料的高效结合,影响了材料的稳定性和应用性能。后续可通过计算机模拟预测磁性COFs的结构,进而发展新型的修饰策略,实现COFs与磁性材料的快速修饰,增加其稳定性;(3)当前的制备方法涉及较多复杂的工艺和有害化学品,不仅增加了实验成本,还不符合环境保护的需求。因此有必要探索和开发绿色、低成本的制备方法,设计合成出具有较高普适性的MCOFs基材料,以实现高灵敏度检测多种污染物的同时降低生产成本和环境影响;(4)开发具有多重功能性的MCOFs以拓宽应用领域,如通过在框架中引入不同官能团使得材料能同时在多个领域(催化、传递药物、富集等)发挥作用;(5)提升MCOFs的抗污染能力,实现长期循环使用、提升经济效益。

## References

[1] Sharma S, Gupta A. Appl Water Sci, 2022, 12(6): 115 35441072 10.1007/s13201-022-01625-3 PMC9010712

[2] Zhou Q, Zhao K, Wu Y, et al. Chemosphere, 2021, 281: 130900 34044305 10.1016/j.chemosphere.2021.130900

[3] Razak M R, Aris A Z, Sukatis F F, et al. J Sep Sci, 2023, 46(1): 2200282 10.1002/jssc.202200282 36337037

[4] Montemurro N, Postigo C, Lonigro A, et al. Anal Bioanal Chem, 2017, 409(23): 5375 28493020 10.1007/s00216-017-0363-1

[5] Su D, Li W, Xu Q, et al. Fitoterapia, 2016, 112: 45 27223850 10.1016/j.fitote.2016.05.004

[6] Ragland J M, Liebert D, Wirth E. Anal Chem, 2014, 86(15): 7696 25007285 10.1021/ac501615n

[7] Xu M L, Gao Y, Wang X, et al. Foods, 2021: 2473 34681522

[8] Zhang Z, Xiao Q, Jiang Z, et al. Rapid Commun Mass Spectrom, 2023, 37(17): e9591 37580507 10.1002/rcm.9591

[9] Wu K S, Xu H Y, Guo L, et al. Food Science, 2011, 32(23): 317

[10] Jiang H L, Li N, Cui L, et al. TrAC-Trends Anal Chem, 2019, 120: 115632

[11] Pang L, Zhang W, Zhang W, et al. RSC Adv, 2017, 7(85): 53720

[12] Guan Y, Pan Y M. Chemical Analysis and Meterage, 2024, 33(4): 90

[13] Timofeeva I, Stepanova K, Bulatov A. Talanta, 2021, 224: 121888 33379097 10.1016/j.talanta.2020.121888

[14] Scigalski P, Kosobucki P. Molecules, 2020, 25(21): 4869 33105561 10.3390/molecules25214869 PMC7659476

[15] Llompart M, Celeiro M, García-Jares C, et al. TrAC-Trends Anal Chem, 2019, 112: 1

[16] Yu H, Zhao Y, Yang L, et al. Anal Methods, 2017, 9(48): 6808

[17] Li N, He Z J, Zhao J H, et al. Colloids Surfaces A Physicochem Eng Asp, 2023, 678: 132431

[18] Corps Ricardo A I, Abujaber F, Guzmán Bernardo F J, et al. Trends Environ Anal, 2020, 27: e00097

[19] Wang X, Feng T, Wang J, et al. J Chromatogr A, 2019, 1602: 178 31301797 10.1016/j.chroma.2019.06.046

[20] Šafaříková M, Šafařík I. J Magn Magn Mate, 1999, 194(1): 108

[21] Zhou Q, Yuan Y, Sun Y, et al. J Chromatogr A, 2021, 1639: 461921 33524931 10.1016/j.chroma.2021.461921

[22] Hao L, Wang Y, Wang C, et al. Microchim Acta, 2019, 186(7): 431 10.1007/s00604-019-3583-6 31187290

[23] Wu A, Zhao X, Wang J, et al. Crit Rev Env Sci Tec, 2021, 51(1): 44

[24] Aghayi-Anaraki M, Safarifard V. Eur J Org Chem, 2020, 2020(20): 1916

[25] Wang Q, Gao T, Hao L, et al. TrAC-Trends Anal Chem, 2020, 132: 116048

[26] Song X L, Lv H, Liao K C, et al. Talanta, 2023, 253: 123930 36113335 10.1016/j.talanta.2022.123930

[27] Ding Z, Zhang K, Yang W, et al. Microchem J, 2023, 195: 109459

[28] Herrero-Latorre C, Barciela-García J, García-Martín S, et al. Anal Chim Acta, 2015, 892: 10 26388472 10.1016/j.aca.2015.07.046

[29] Trujillo-Rodríguez M J, Pino V, Anderson J L. Talanta, 2017, 172: 86 28602308 10.1016/j.talanta.2017.05.021

[30] An J, Rahn K L, Anderson J L. Talanta, 2017, 167: 268 28340720 10.1016/j.talanta.2017.01.079

[31] Zhou D D, Cao Y W, Chen M, et al. Microchem J, 2023, 187: 108459

[32] Chen X, Ding N, Zang H, et al. J Chromatogr A, 2013, 1304: 241 23880470 10.1016/j.chroma.2013.06.053

[33] Mi X, Zhou S, Zhou Z, et al. Colloids Surfaces A: Physicochem Eng Aspects, 2020, 603: 125238

[34] Chen Y, Chen Z. Talanta, 2017, 165: 188 28153241 10.1016/j.talanta.2016.12.051

[35] Liu Z, Wang J, Duan T, et al. J Chromatogr A, 2022, 1679: 463387 35933771 10.1016/j.chroma.2022.463387

[36] Wang N, Zhou X, Cui B. J Chromatogr A, 2023, 1687: 463702 36508770 10.1016/j.chroma.2022.463702

[37] Zhang S, Yang Q, Wang C, et al. Adv Sci, 2018, 5(12): 1801116 10.1002/advs.201801116 PMC6299720 30581707

[38] Torabi E, Mirzaei M, Bazargan M, et al. Anal Chim Acta, 2022, 1224: 340207 35998988 10.1016/j.aca.2022.340207

[39] Yang C, Mo Z, Zhang Q, et al. Food Chem, 2024, 438: 137984 37979275 10.1016/j.foodchem.2023.137984

[40] Zhang S, Wang R, Wu Y, et al. J Chromatogr A, 2022, 1667: 462906 35202922 10.1016/j.chroma.2022.462906

[41] Zhang W, Liang F, Li C, et al. J Hazard Mater, 2011, 186(2): 984 21159428 10.1016/j.jhazmat.2010.11.093

[42] Wang W, Shao H, Sun C, et al. Environ Sci Nano, 2022, 9(4): 1466

[43] Wang J, Li J, Gao M, et al. Nanoscale, 2017, 9(30): 10750 28715013 10.1039/c7nr02932b

[44] Wang Y F, Mu G D, Wang X J, et al. Microchim Acta, 2021, 188(8): 246 10.1007/s00604-021-04893-z 34235593

[45] Yao L, Zhang J, Xia J, et al. Microchem J, 2024, 200: 110283

[46] Gao Y Y, Ding Y L, Chen L Y, et al. Chinese Journal of Chromatography, 2023, 41(7): 545 37387275 10.3724/SP.J.1123.2022.12021 PMC10311619

[47] Wang J, Zhuang S. Coord Chem Rev, 2019, 400: 213046

[48] Li X, Kawai K, Fujitsuka M, et al. SurfInterfaces, 2021, 25: 101249

[49] Coté A P, Benin A I, Ockwig N W, et al. Science, 2005, 310(5751): 1166 16293756 10.1126/science.1120411

[50] Fu X R. Biochemical Engineering, 2020, 6(5): 135

[51] Patial S, Soni V, Kumar A, et al. Environ Res, 2023, 218: 114982 36495966 10.1016/j.envres.2022.114982

[52] Yang X S, Wang L L, Liu Y S, et al. Food Chem, 2022, 386: 132843 35381536 10.1016/j.foodchem.2022.132843

[53] He S, Zeng T, Wang S, et al. ACS Appl Mater Interfaces, 2017, 9(3): 2959 28075557 10.1021/acsami.6b13643

[54] Yu L, Xia A, Hao Y, et al. Molecules, 2024, 29(10): 2311 38792172 10.3390/molecules29102311 PMC11123868

[55] Wen A, Li G, Wu D, et al. J Chromatogr A, 2020, 1612: 460651 31753482 10.1016/j.chroma.2019.460651

[56] Pang Y H, Yue Q, Huang Y Y, et al. Talanta, 2020, 206: 120194 31514904 10.1016/j.talanta.2019.120194

[57] Fu J G, Xie L X, Du J, et al. ACS Sustain Chem Eng, 2024, 12(37): 13873

[58] Deng Z H, Wang X, Wang X L, et al. Microchim Acta, 2019, 186(2): 108 10.1007/s00604-018-3198-3 30637544

[59] Zhao D, Xu X, Wang X, et al. Microchim Acta, 2023, 190(12): 488 10.1007/s00604-023-06051-z 38015320

[60] Wang R, Chen Z. Microchim Acta, 2017, 184(10): 3867

[61] Lin X, Wang X, Wang J, et al. Anal Chim Acta, 2020, 1101: 65 32029120 10.1016/j.aca.2019.12.012

[62] Li J, Xu X, Wang X, et al. Microchim Acta, 2022, 189: 149

[63] Liu J, Li G, Wang P. Microchem J, 2022, 174: 106987

[64] Xu G, Hou L, Liu C, et al. ACS Appl Mater Interfaces, 2021, 13(43): 51535 34672528 10.1021/acsami.1c15869

[65] Li Y, Zhang H, Chen Y, et al. ACS Appl Mater Interfaces, 2019, 11(25): 22492 31180623 10.1021/acsami.9b06953

[66] Liao L, Li M, Yin Y, et al. ACS Omega, 2023, 8(5): 452 10.1021/acsomega.2c06961 PMC9909813 36777586

[67] Ma J Q, Ren J Y, Wang L L, et al. J Sep Sci, 2018, 41(19): 3724 30088340 10.1002/jssc.201800630

[68] Shi X, Li N, Wu D, et al. Anal Methods, 2018, 10(41): 5014

[69] Yan Y, Lu Y, Wang B, et al. ACS Appl Mater Interfaces, 2018, 10(31): 26539 30016867 10.1021/acsami.8b08934

[70] Gao C, Bai J, He Y, et al. ACS Appl Mater Interfaces, 2019, 11(14): 13735 30892013 10.1021/acsami.9b03330

[71] Luo B, He J, Li Z, et al. ACS Appl Mater Interfaces, 2019, 11(50): 47218 31750645 10.1021/acsami.9b15905

[72] Gao C, Bai J, He Y, et al. ACS Sustain Chem Eng, 2019, 7(23): 1892

[73] Wang Z, Zou T, Feng S, et al. Anal Chim Acta, 2023, 1278: 341691 37709444 10.1016/j.aca.2023.341691

[74] Liang R, Hu Y, Li G. J Chromatogr A, 2020, 1618: 460867 31959461 10.1016/j.chroma.2020.460867

[75] Zhou J, Xu D, Cao J, et al. Foods, 2024, 13(3): 409 38338544

[76] Jiang L, Zhang Y, Huang S, et al. J Clean Prod, 2024, 439: 140910

[77] Li N, Wu D, Li X, et al. Food Chem, 2020, 306: 125455 31629968 10.1016/j.foodchem.2019.125455

[78] Sun W, Xu Q, Liu Q, et al. J Chromatogr A, 2023, 1690: 463777 36640681 10.1016/j.chroma.2023.463777

[79] Zheng Z, Xu K, Lu F, et al. Environ Sci Pollut Res, 2023, 30(12): 34636 10.1007/s11356-022-24720-z 36515884

[80] Zhang X, Li F, Chao J, et al. Anal Chim Acta, 2024, 1307: 342622 38719403 10.1016/j.aca.2024.342622

[81] Zhuang S, Wang J. Chemosphere, 2021, 264: 128561 33049505 10.1016/j.chemosphere.2020.128561

[82] Ge J, Xiao J, Liu L, et al. J Porous Mater, 2016, 23(3): 791

[83] Lin S, Lv Y K, Zhu A, et al. Food Chem, 2024, 454: 139796 38797102 10.1016/j.foodchem.2024.139796

[84] Niu L, Zhao X, Tang Z, et al. Sep Purif Technol, 2022, 294: 121145

[85] Liu G, Chen H, Zhang W, et al. Anal Chim Acta, 2021, 1166: 338539 34022997 10.1016/j.aca.2021.338539

[86] Biswal B P, Chandra S, Kandambeth S, et al. J Am Chem Soc, 2013, 135(14): 5328 23521070 10.1021/ja4017842

[87] Li S, Liu W, Wang Q, et al. Food Chem, 2022, 382: 132362 35152018 10.1016/j.foodchem.2022.132362

[88] Guo H, Li Y, Li Y, et al. ACS Appl Mater Interfaces, 2023, 15(11): 14777 10.1021/acsami.2c22386 36897016

[89] Fan J, Liu Z, Li J, et al. J Chromatogr A, 2020, 1619: 460950 32061359 10.1016/j.chroma.2020.460950

[90] Chen L, Chen B, Zhou Z, et al. J Chromatogr A, 2022, 1673: 463030 35487115 10.1016/j.chroma.2022.463030

[91] Guo L, Liu J, Li J, et al. J Chromatogr A, 2021, 1651: 462301 34107399 10.1016/j.chroma.2021.462301

[92] Tian C, Wu Z, He M, et al. J Sep Sci, 2022, 45(10): 1746 35218314 10.1002/jssc.202200055

[93] Li N, Wu D, Hu N, et al. J Agric Food Chem, 2018, 66(13): 3572 29554797 10.1021/acs.jafc.8b00869

[94] Zhang W, Lan C, Zhang H, et al. J Agric Food Chem, 2019, 67(13): 3733 30835454 10.1021/acs.jafc.8b06372

[95] Wang J, Huang Q, Guo W, et al. Toxins, 2023, 15(2): 117 36828431 10.3390/toxins15020117 PMC9966527

[96] Wang Y X, Zhang W, Shen X F, et al. Anal Methods, 2023, 15(9): 1135 36779345 10.1039/d2ay01989b

[97] Liang M, Li N, Zhang H, et al. RSC Adv, 2024, 14(13): 8726 38500629 10.1039/d4ra00496e PMC10945740

[98] Cheng Y N, Ding T D, Qian Y G, et al. Chinese Journal of Biotechnology, 2019, 35(11): 2151 31814361 10.13345/j.cjb.190191

[99] Ning T, Di S, Li Z, et al. Anal Chim Acta, 2023, 1239: 340615 36628698 10.1016/j.aca.2022.340615

[100] Huang L, Shen R, Liu R, et al. Food Chem, 2021, 347: 129002 33482486 10.1016/j.foodchem.2021.129002

[101] Li N, Liang M, Zhang H, et al. RSC Adv, 2024, 14(12): 8303 38487520 10.1039/d4ra00247d PMC10938296

[102] Gao J, Ouyang J, Shen J, et al. J Chromatogr A, 2023, 1706: 464283 37562103 10.1016/j.chroma.2023.464283

[103] Ma L, Gu Y, Guo L, et al. RSC Adv, 2024, 14(30): 21318 38979455 10.1039/d4ra02530j PMC11228574

